# Who Needs Values When We Have Valuing? Comments on Jean Moritz Müller, *The World-Directedness of Emotional Feeling*

**DOI:** 10.1177/17540739221085575

**Published:** 2022-03-07

**Authors:** Ronald de Sousa

**Affiliations:** University of Toronto

**Keywords:** axiological receptivity, position-taking, attitudinalism, value

## Abstract

Müller argues that the perceptual or “Axiological Receptivity” (AR) model of emotions is incoherent, because it requires an emotion to apprehend and respond to its formal object at the same time. He defends a contrasting view of emotions as “Position-Takings" (PT) towards “formal objects”, aspects of an emotion's target pertinent to the subject's concerns. I first cast doubt on the cogency of Müller's attack on AR as begging questions about the temporal characteristics of perceptual events. I then argue that Müller's version of PT is not radical enough. On my attitudinal view, formal objects are not values but natural properties that justify specific affective or behavioral responses. Values are constituted only by a negotiated social aggregation of individual evaluative attitudes.

Müller (2017) frames his exposition as a contest between two broadly defined conceptions of the relation between emotions and values: Axiological Receptivity (AR) and Position-Taking (PT). Towards the end of Müller's book, I was startled to read a footnote that made me wonder whether I myself have not held both views: “It seems to me”, Müller wrote, “that there is a question how close de Sousa's claim that emotions are attitudes is to the idea of a position-taking. As I understand it, it is principally aimed at capturing the idea that emotions are perspectival in that one cannot hypothetically experience them” (Müller, 2017, p. 108). This sounds right: in the book he refers to (i.e. de Sousa, 1987), despite my use of the word “attitude”, I aired an analogy between emotions and perception. I am now glad of the opportunity to come down firmly on the side of an attitudinal view very similar to PT. My current attitudinal view, however, differs from Müller's in two ways. Before I explain what these are, I want to express some reservations about Müller's phenomenological method, and about the cogency of the argument against AR that relies on it.

## Limitations of the Phenomenological Method

Müller (2017) writes that his “arguments will be primarily based on the way emotional feelings present themselves from a first-person point of view—their phenomenology—and the linguistic and conceptual structures we deploy in ascribing and making sense of them.” (p. 10). This perspective plays an important role in his criticism of AR. And while I am far from confident that I understand phenomenology as a philosophical method, I think that late 20th Century work in philosophy of mind should make us suspicious of the quest for essences grounded in what is accessible to the first-person point of view. Hilary Putnam (1973) has persuasively argued that “meanings ain’t in the head”; Ruth Millikan (1993) has attacked “meaning rationalism”, the doctrine that we have incorrigible access to meaning identity, univocity, and meaningfulness. The meaning of a term in my idiolect is determined by a complex history, of which I could not possibly be aware. This makes me uneasy about Müller's reliance on what he calls the “manifest image” of emotion in his rejection of AR.

## The Negative Argument Against AR

AR “conceives of [emotion] as a form of impression that constitutes direct epistemic contact with value.” (Müller, 2017, p. 52). It is best exemplified by perceptual theories, such as that of Christine Tappolet (2016), which regard emotions as apprehensions of value. Perceptual views face a number of objections, such as the absence of a sensory channel in emotions, and the implausibility of applying rational standards in sensory perception. To these Christine Tappolet has provided answers (Tappolet, 2016), which I will not discuss. Müller's core objection is more radical: AR is incoherent, because an emotional episode presupposes the very apprehension of value it is supposed to consist in:

the appearance [of value] temporally precedes the feeling. Accordingly, in such cases, the respective value is apprehended prior to the feeling. But this means that the feeling does not apprehend or disclose this value: one cannot apprehend what one has already apprehended. (Müller, 2017, p. 72)

Müller puts the argument formally as follows:

1. Necessarily, the felt aspect of S's emotion responds to how its target x evaluatively appears to S.

2. Where S's emotional feeling towards x is a response to how x appears to S, the appearance temporally precedes S's feeling.

3. In some cases, the prior appearance constitutes an apprehension of the value of x; in the other cases, it constitutes a mere apprehension as of the value of x.

4. When S's emotional feeling responds to an appearance which apprehends the value of x, S apprehends this value prior to S's feeling.

5. It is not possible for S to apprehend what S has already apprehended.

6. When S's emotional feeling is responsive to an appearance which apprehends the value of x, S's feeling does not apprehend the value of x. (Müller, 2017, p. 72).

Therefore, Müller concludes:

emotional feelings cannot be disclosive of value when they are at the same time based on awareness of value. … If for them to be directed implies that they are responses to apparent value, then emotional feelings do not present or otherwise apprehend value. (Müller, 2017, pp. 72–73).

Premises (1) and (3) are unobjectionable. But in (5), apprehension must be understood in an unusual way, as an instantaneous event. Apprehension is more naturally understood as a process, resulting in a state that can vary in clarity, detail, and valence. We cannot assume that it just switches from *off* to *on* at a single instant. *As I took in the scene,* we sometimes say, *I realized that it was scary*. Thus (5) is false, unless “apprehension” is restricted to the instantaneous onset of acquaintance. When restricted thus, (5) becomes a tautology, which doesn’t advance the argument. Either way, the argument fails.

The point can be made by pointing out that premises (2) and (4) beg questions of temporality. What exactly is the force of the conclusion that “disclosure of value” and “awareness of value” occur (or cannot occur) “*at the same time*”? AR aims to capture the intuition that emotional episodes make salient certain aspects of a target situation (or person, or fact) affecting the subject's concerns. That does not entail that an emotional episode must be instantaneous. Like perception, to which AR assimilates it, emotion is a process that results in a certain state of awareness. To be sure, a red *quale* may seem to just present itself. But it is not “semantically anomalous” (to borrow a term Müller uses) to say that my experience of red *constitutes my visual system's response* to the red stimulus. Vision is a complicated process that unfolds in time, albeit largely below the threshold of awareness. As a recent book on color vision puts it,

[D]espite the compelling salience of the perceived environmental colors we may experience while appreciating a magnificent natural scene, color experience should not be thought of as the act of simply registering features that exist in the world. Nor should it be thought of as an unbiased personal index of object properties. Rather, color is a product of observers’ minds, existing as highly individualized constructions of each observer's visual apparatus and the specific ways it translates sensory information received from the world. (Jameson et al., 2020, p. 2).

These processes are not instantaneous, however they may appear. Complex computations are involved, notably of certain ratios of responses between different kinds of retinal cones; these must be fed into, and thus precede, the visual cortex's processing which gives rise to the experienced *quale* (Churchland & Churchland, 1998, 166–172). The former are not conscious, but they are essential to the mechanism that produces the conscious *quale*. They are part of the seeing event.

Similarly, I see no incoherence in the supposition that the feeling of fear involves a process that as a whole *just is* what apprehension of a value (or of the instantiation of a formal object) consists in. In fact, there are three ways in which AR might explain this, and thus evade Müller's charge of incoherence.

First, we might say that the apprehension-of-and-response-to a value property, just like the process of apprehending a visual *quale* just described, is caused by processes that occur at the sub-personal level. These must take time, despite appearing instantaneous. We know from Robert Zajonc that “preferences need no inferences” (Zajonc, 1980, 2000), meaning that the apprehension of an object can be felt as immediately valenced independently of any other cognitive state. The mere fact that a pattern has been seen before, without awareness at the time or feeling of recognition when presented again, can be sufficient to influence liking. Valence, then, requires no prior apprehension of *qualia*; but that does not require that there be no sub-personal processes that produced it. It simply means that awareness does not extend to the causal processes that result in that experience of liking.

Second, we might, with appraisal theories, regard emotions as either caused or constituted by appraisals in several dimensions. Agnes Moors and others have debated the temporal or causal priority of appraisal versus conscious emotion (Moors, Ellsworth, Scherer, & Frijda, 2003; Moors, 2013). Whether appraisals constitute the essence of emotion, or cause emotions’ other components, is at least in part amenable to empirical investigation, rather than being directly revealed by phenomenology.

A third reply might be inspired by Uriah Kriegel's taxonomy of irredudible kinds of conscious states. Kriegel suggests that emotional qualia, if not themselves primitive, consist in episodes of “proprioceptive, algedonic, cognitive, and conative” modes of consciousness (Kriegel, 2015, p. 147). If so, then, even from a purely phenomenological point of view, it might be that a *valenced quale* constitutes an irreducibly primitive species of apprehension. The claim that it must involve two temporally distinct episodes would simply beg the question.

In the light of these possible responses, Müller's core objection to AR does not seem compelling. But he has a second objection, based on the epistemic role that AR is commonly taken to play.

AR is primarily concerned with occurrences of emotion that are appropriate or fitting. Strictly speaking, the version of intentionalism which I am here examining conceives of the felt aspects of emotional occurrences as presentations of value only when the emotion is appropriate or fitting. (When it is not fitting, emotional feelings are thought to constitute mere presentations *as of* value. … Thus, when I say that AR understands emotional feelings as epistemically significant, it is really the feelings involved in fitting emotions which are accorded epistemic significance.) (Müller, 2017, p. 54).

What “epistemic significance” actually amounts to isn’t entirely obvious; but it seems reasonable to take it as referring to the character of my emotion as informative about the target I am responding to. An emotion provides evidence of the instantiation of its formal object. Thus understood, the passage just quoted says that, according to AR, my emotion cannot be taken to provide evidence for *p* until I know that *p* is actually true. But surely I can judge whether some fact is or is not evidence for *p* without knowing whether *p* is true. This would indeed be a problem for AR, if AR were necessarily committed to it.

But I see no reason to think it is. I can take my fear as evidence that the target is dangerous even if it turns out to be inapt. Misleading evidence is still evidence.

## A Slightly Different Position on Position-Taking

Despite my doubts about Müller's refutation of AR, I have come to prefer an attitudinalist view that is very close to his. As he notes, “attitude” and “position-taking” in this context are more or less equivalent (Müller, 2017, p. 8, fn 10); I will continue, however, to speak of “attitudes” rather than “position-takings”. My reason is that in my idiolect “position-taking” (like *prise de position* and *Stellungnahme*) connotes intentionality (in the *other* sense of that word, connoting voluntary control). That seems to misrepresent the passivity that characterizes typical emotional phenomenology (Gordon, 1986). Furthermore, while I can make sense of taking a position in relation to a value, I would most naturally interpret talk of *taking a position with respect to a value* as expressing a meta-level (emotional or cognitive) attitude. Towards the value of chastity, for example, I take an emotional attitude of disapproval, regarding its observance as harmful. In a purely descriptive mode, I also take a cognitive attitude, believing that it served to cement men's control of women in patriarchal societies.

Aside from this terminological nicety, I have just one substantive disagreement with Müller. It is that he is not radical enough. Before I explain, let me line up a few sentences with which I am in agreement, building up to the point of divergence.

- Emotions are emotional states with a characteristic phenomenology: “an adequate view of emotions ought to recognize them as having a specific felt aspect”. (Müller, 2017, pp. 2–3).
- “Emotions make a distinctive contribution to our mental lives qua feeling” (Müller, 2017, p. 3). I interpret “qua feeling” as referring to the character of the emotional attitude as determined by its formal object.
- “feeling towards is a response to certain aspects of its object” (Müller, 2017, p. 51).
- “PT will be understood as conceiving of emotional feelings as responses to their formal object” (45–6).
- Emotion “constitutes the taking of a positive or negative position on some object or event. . . . where this position is crucially informed by our personal investments, that is, by our cares and concerns” (Müller, 2017, p. 8).
- A formal object is a “specific type of . . . property which plays an essential role in determining their intelligibility, fittingness and individuation conditions” (Müller, 2017, p. 11). Thus sadness is a response to loss; fear is response to danger; anger is a response to offense.
  On all these, we agree. So where do we differ?

The answer is to be found in one word I omitted from the last two quotations. The word is *value.* On p. 11, the “property which plays an essential role…” is a “*value* property”. On p. 8, the lacuna reads “in response to its value”. Müller repeatedly stresses this point: “the formal objects of emotions are response-independent axiological properties.*”* (p. 38.)

By regarding the formal object to be a *value*, or *axiological property,* Müller's version of PT seems to undermine what I regard as his central contention: “feeling towards is a *response* to certain aspects of its object.” (Müller, 2017, p. 12, emphasis in the text). The main point of moving away from AR, as I conceive it, is to locate the normative character of the emotion in the response, rather than in the target.

Consider the pain that arises from putting your finger on a flame. The heat is in the flame, but the pain is a response to it. Though we might say the flame is *painful,* the disvalue is located in our response, not in the flame itself. Similarly, my emotions-as-attitudes are just directed at the things, persons, situations, or states of affairs to which they are responding. Müller's emotions-as-position-takings, by contrast, are directed at values. But that is both obscure and redundant. We agree that the emotion does not *detect* value. As we saw, however, Müller requires the subject to *have detected* value before responding to it. But by what faculty do we detect value? Of that he says nothing. For AR, “detecting a value” *includes being affected* by it. For Müller, the “being affected” is a separate, posterior event. But since it is, if apt, determined by the pre-detected “value”, it seems redundant.

To put it differently, our disagreement lies in the ways Müller and I construe the notion of formal object. For me, the formal object is not an axiological property: it is the natural property on which my response confers “value”. Danger, for example, is the objective likelihood of harm, to me or what I care about, that rationalizes my attitude. It does so by making intelligible the “action-readiness” which is the basis of my emotional feeling (Deonna & Teroni, 2015, p. 302). Dangerousness, though hard to define precisely, and obviously relative to my interests and concerns, is a non-normative, response-independent property. It is not contingent on my subjective attitude. By contrast, the value property of the *frightening* or *fearsome*, which is often said to supervene on the dangerous (Müller, 2017, p. 136), is just the shadow projected by that attitude of fear. It is experienced as a property possessed by the target, in virtue of the fact that the dangerousness can be apprehended as providing an adequate cognitive basis for my attitude of fear. That is what makes it apt. If I find a target frightening despite not being dangerous, my fear is inapt. Thus it is the dangerous, not the value property of the fearsome, that fulfills the formal object's function of “correctness” condition. AR is really about the psychological reality of an illusion; what made it plausible is that our emotions project values onto their targets in virtue of being the emotional attitudes they are.^1^

## More About Responding to Value

The view that my response *constitutes*, as opposed to being directed at, value, may seem paradoxical. A liking, preference or pro attitude is not yet a value. What more is needed? Does an attitude make a value only when several people share it? How many people do we need to value something in order to justify calling it valuable? Could there be values that no one responds or ever has responded to? These are all good questions for value theory, but they give us no reason to reject the attitudinal view.

Birds, bees (and human tetrachromats; see Jameson et al., 2020) see colors the rest of us can’t see. Are there, similarly, values we don’t respond to with apt attitudes? A positive answer could rest on two observations. I can have a dispositional attitude that on a particular occasion is not manifested in an occurrent emotion. More importantly, one might insist on reserving the word “value” for assessments that in some way have become public, or common (though not necessarily universal). This could result from a kind of negotiation, on a societal scale, among expressions of individual response. This raises questions about when my individual emotional response can and when it cannot be described as expressing a value. On my version of attitudinalism, my attitude is justified (or fails to be justified, if it is inapt) by the detection in the target of natural properties that make sense of the appropriate action-readiness entailed by my emotion. That justification may depend on all kinds of factual information, including inferences from physics and biology. Suppose, for example, that I am moved by fear to elude the predator that is the target of my fear. My position-taking as a state of fear will involve an action readiness aiming at eluding the dangerous predator. On the basis of the exchange of similar stories, I may find that my particular attitude to dangerous situations is widely shared in my linguistic community. As a consequence of talking about it, what started as a great many individual responses to certain situations and things occurring in the world will coalesce into a value of *the frightening,* generally recognized by us.^2^ But who are *we*?

The elusive character of this last question is concealed behind the comfortable assumption that at least some of the targets of our emotions exemplify “response-independent” values. But if the frightening, the sad, the disgusting, etc. are just shadows of attitudes taken by a number of members of a linguistic group towards certain types of targets, the implied anti-realism about axiological properties affects our conception of the world and our relation to it. Logically and psychologically, any attitude *could* be taken to any fact or situation. “It is not contrary to reason for me to chuse my total ruin, to prevent the least uneasiness of an Indian or person wholly unknown to me.” (Hume, 1978, Bk 2-Pt III §iii). Independently of our “concerns”, the world is devoid of both values and value. That, it seems to me, is the true lesson of Müller's view of emotions as position-takings, but it is obscured by his insistence that formal objects are *values* in any sense beyond being *valued*. The thought that value is conferred by an elusive “us”, rather than waiting to be detected-and-responded-to, raises a number of questions about who *we* are, how much consensus is required to turn individual pro-attitudes into values, and just what natural facts justify what attitudes. These are important questions that should be addressed; but not here, now, by me.

## Notes

1. In this regard, my view is also more radical than that of Deonna and Teroni (2012, 2015) who, like Müller, regard the attitude or position-taking as directed towards a value property. I have no space for any discussion of the nuances between different versions of attitudinal theories. Müller distances himself from Deonna and Teroni (2012, 2015) by denying that any properioceptive sensations related to action-readiness are part of the phenomenological experience of emotion (Müller 2019, 23–4); he also claims that Deonna and Teroni “misunderstand the evaluative character of emotions…. emotions are not ways of coming to be aware of value, but ways of *acknowledging* values of which we are already aware.” (Müller, 2017, p. 282). Insofar as I am able to understand that distinction, my own view is closer to that of Deonna and Teroni.

2. For some emotions, to be sure, there will be no distinct action-readiness by which they can be uniquely identified. Examples are what Tappolet calls “contemplative emotions” (Tappolet, 2016, pp. 64–76). This suggests that some values are more objective than others. (See de Sousa, 2017).
